# A female-biased sex ratio reduces the twofold cost of sex

**DOI:** 10.1038/srep23982

**Published:** 2016-04-01

**Authors:** Kazuya Kobayashi, Eisuke Hasegawa

**Affiliations:** 1 Laboratory of Animal Ecology, Department of Ecology and Systematics, Graduate School of Agriculture, Hokkaido University, Sapporo 060-8589, Japan

## Abstract

The evolution of sexual reproduction remains a fascinating enigma in biology. Theoretically, populations of sexual organisms investing half of their resources into producing male offspring that don’t contribute to reproduction should grow at only half the rate of their asexual counterparts. This demographic disadvantage due to male production is known as the twofold cost of sex. However, the question of whether this cost is truly twofold for sexual females remains unanswered. The cost of producing males should decrease when the number of male offspring is reduced. Here, we report a case where the cost of males is actually less than twofold. By measuring the numbers of sexual strain coexisting with asexual strain among thrips, our survey revealed that the sexual strain showed female-biased sex ratios and that the relative frequency of sexual strain is negatively correlated with the proportion of males in the sexual strain. Using computer simulations, we confirmed that a female-biased sex ratio evolves in sexual individuals due to the coexistence of asexual individuals. Our results demonstrate that there is a cost of producing males that depends on the number of males. We therefore conclude that sexual reproduction can evolve with far fewer benefits than previously assumed.

The dominance of sexual reproduction in nature is one of the most prevalent enigmas in evolutionary biology. Most sexual organisms invest half of their resources into producing male offspring, which do not contribute to population growth^1^. As a result, populations of sexual organisms grow at only half the rate of their asexual counterparts^2^,^3^. Even with this twofold cost of producing male offspring, sexual reproduction is common in nature, and most organisms engage in sexual reproduction. Therefore, the benefit of sexual reproduction should be large for it to have not only evolved but also persisted in most organisms. Consequently, numerous studies have been conducted to investigate the benefits of sexual reproduction^4^,^5^,^6^,^7^,^8^,^9^,^10^. By contrast, few studies have attempted to identify the cost of male production^11^,^12^,^13^,^14^,^15^. Even in the studies that do focus on such costs, sex ratios are assumed to be fixed. However, there is a possibility that female-biased sex ratios evolved in sexual individuals to compete with their asexual counterparts. This possibility has been overlooked because sex ratio theory predicts that female-biased sex ratios contributing to population growth should be eliminated by individual selection, leading to equal sex ratios. Here, we show that the cost of male production is actually less than twofold in a sexual strain of the onion thrips *Thrips tabaci*.

Sexual and genetically isolated asexual strains of onion thrips (*T. tabaci*) coexist on a single host plant^16^,^17^. These strains are almost identical in both their morphology and ecology and are only identifiable by genetic markers^16^,^17^,^18^,^19^,^20^. Because sex ratios vary among populations of the sexual strain^16^,^17^, we can ascertain the cost of producing male offspring by exploring the frequencies of sexual strain with various sex ratios. In *T. tabaci*, the sexual strain should be able to increase their population growth rate by reducing the frequency of male offspring, thus resulting in increased competitiveness against coexisting asexual strain.

In this study, we evaluated the relationship between the sex ratio in sexual strain and the sexual:asexual ratio in *T. tabaci* from 32 crop fields in Hokkaido, Japan. Moreover, we investigated two possible mechanisms of maintaining female-biased sex ratios in sexual strain. One is an effect of local mate competition (LMC)^21^ among sexual strain, which was analyzed based on the degree of inbreeding in the sexual strain. The other is an effect of pesticide applications causing local extinction. Because *T. tabaci* is an economically important pest insect, farmers respond to a high density of thrips with pesticide application before crop (resource) exhaustion^17^. Under these conditions, sexual strain is forced to maintain a high reproductive rate by maintaining female-biased sex ratios or to be extinct. We tested this pesticide hypothesis with a simulation model. To confirm the validity of the simulation parameters, we explored the reproductive rates of sexual and asexual strains under laboratory conditions, and the effect of distance from the sea on the sexual:asexual ratio as a proxy for temperature fluctuations, which should be beneficial to the sexual strain.

## Results and Discussion

**Figure 1:**
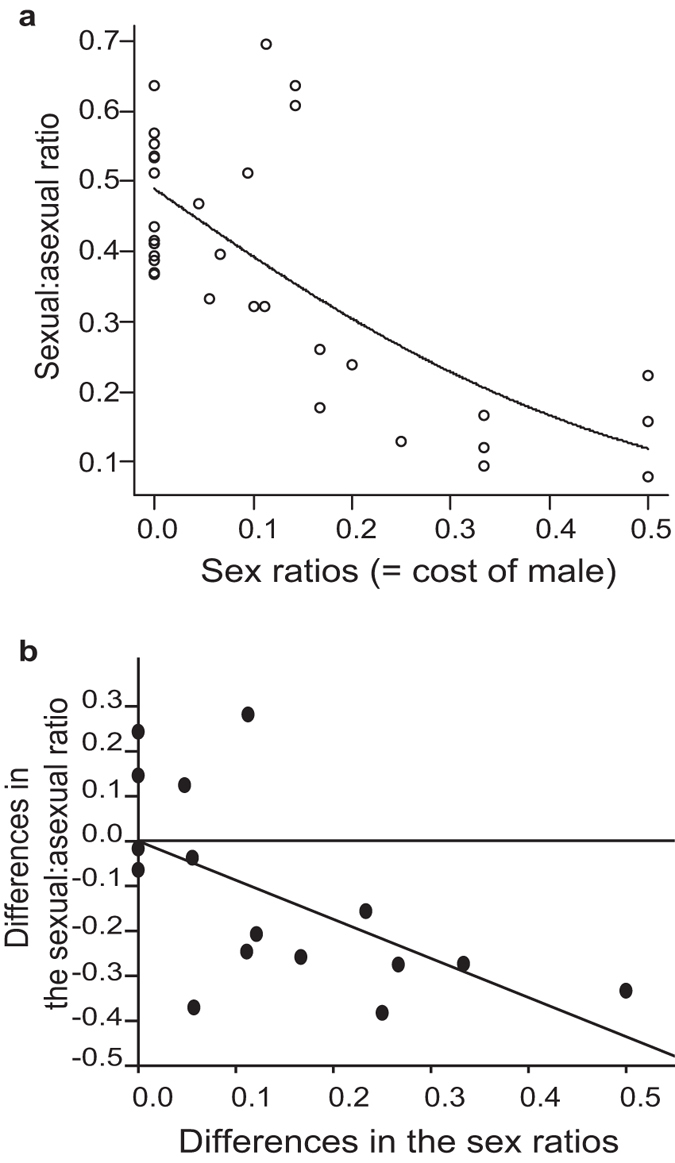
The sexual strain dominates in a population with a female-biased sex ratio. (**a**) The effect of the sex ratio (proportion of males in sexual strain) on the sexual:asexual ratio (proportion of sexual strain in *T. tabaci*). Each data point represents a population. (**b**) The effect of changes in the sex ratio on the changes in the sexual:asexual ratio over evolutionary history. The data were evaluated from 16 phylogenetically independent pairs of the populations.

Our field survey showed that sex ratios were significantly biased toward females in many populations of the sexual strain (17 of the 32 populations, *p* < 0.05 based on binomial tests after Bonferroni corrections). Here, the sexual:asexual ratios were negatively correlated with the sex ratios (Fig. 1a; generalized linear model (GLM); *n* = 32 populations; likelihood ratio *x*^*2*^ = 26.22, *p* < 0.001; Pearson’s product-moment correlation, *r* = −0.709, *p* < 0.001). In populations with a high cost of male production (equal sex ratio), there were almost no sexual strain. However, in those with a female-biased sex ratio, the number of sexual strain approached the number of asexual strain. Because phylogenetic relationships among populations often lead to statistically erroneous conclusions during correlation analysis^22^, we removed the phylogenetic effect using an independent contrast method by creating a neighbor-joining tree of the populations based on genetic distances (*F*_*ST*_) (see Method and Supplementary Fig. S1 for details). Even after this treatment, the coefficient was significantly negative (Fig. 1b; linear regression analysis without intercept; *n* = 16 independent pairs; *F*_*1,15*_ = 15.88, *p* = 0.001; Pearson’s product-moment correlation, *r* = −0.613, *p* = 0.012).

**Figure 2:**
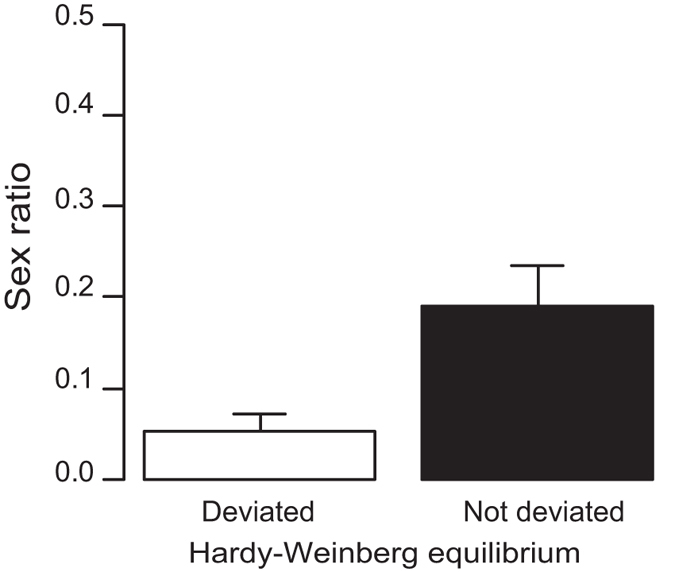
The effects of inbreeding on the sex ratio (proportion of males in sexual strain) among the assessed populations. The 32 evaluated populations were divided into two groups: populations that significantly deviated from Hardy-Weinberg equilibrium (left; *n* = 17) and those that did not (right; *n* = 15). The bars show the mean values and standard errors that did not overlap with equal ratios.

The current female-biased sex ratios of the sexual strain may be explained by LMC^21^. Female-biased sex ratio is favored if mating competition takes place between male offspring^21^, whereas an equal sex ratio is expected under random mating^1^. We therefore examined the effect of LMC on the current female-biased sex ratios. LMC is usually accompanied by inbreeding because males must compete with brothers for mates when mating partners are limited to kin. Thus, we can examine the effect of LMC by examining the relationship between inbreeding coefficients and sex ratios. We found that the inbreeding coefficients were positive in all populations of the sexual strain (Supplementary Table S1). However, we did not detect any significant difference in the sex ratios of sexual strain between the populations deviated and not deviated from Hardy-Weinberg equilibrium (Fig. 2; GLM, *n* = 32 populations; likelihood ratio *x*^2^ = 1.793, *p* = 0.181; Pearson’s product-moment correlation, *r* = −0.314, *p* = 0.080).

Another possible explanation for the female-biased sex ratios in the sexual strain is that they represent an evolved counter-adaptation to the frequent pesticide applications invoked by the invasion of asexual strain. Under this mechanism, the sex ratios of sexual strain are subject to two types of selection pressures: maximization of the genetic contribution within a mating group and selection for population growth rates induced by the pesticide applications. In populations of sexual organism with female-biased sex ratios, the genetic contribution of the sons to the next generation becomes larger than that of the daughters, and selection therefore favors the production of more sons by mothers^1^. By contrast, assuming that the pesticide applications cause local extinctions, mothers that produce more daughters are more likely to avoid extinction. Moreover, the invasion of an asexual strain with a high population growth rate increases the frequency of pesticide application. Hence, the sexual strain is forced to maintain a high population growth rate, comparable to that of the asexual strain, or face demographic extinction. Thus, this type of inter-strain competition may allow the evolution of female-biased sex ratios in sexual strain.

**Figure 3:**
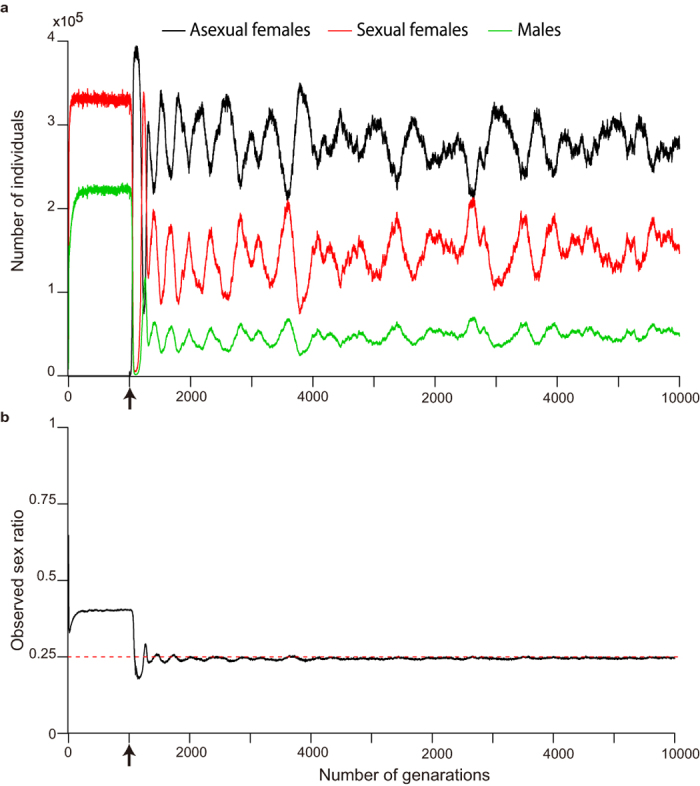
Evolution of female-biased sex ratios in sexual individuals after the invasion of asexual individuals. (**a**) The population dynamics of males and sexual and asexual females are represented by green, red and black lines, respectively. (**b**) The observed sex ratios (proportion of males in a sexual population) are shown with a red dashed line; the value of 0.25 indicates where the growth rate of a sexual population is equal to that of an asexual population. The arrows indicate the time point at which asexual individuals invaded the population. A typical result from a single run is shown, in which the number of offspring per sexual female was 20, and that for asexual female was 15. The probability of dispersal success is 0.1, and the threshold value for pesticide application is 10 individuals per patch.

**Figure 4:**
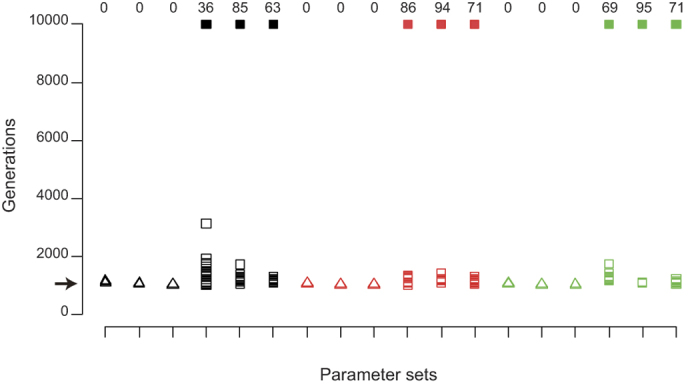
The effect of the simulation parameters on the coexistence of sexual and asexual individuals. The y-axis represents the simulated generations until the extinction of either sexual or asexual individuals occurs (open points), or until 10,000 generations are achieved (filled points). Each column corresponds to each parameter set. The numbers at the top of each column indicate the number of simulations in which coexistence until 10,000 generations is achieved within 100 simulation runs. The different shapes represent the number of offspring per female, which was 20 (triangle) or 15 (square) for asexual females and 20 for sexual females. Thus, sexual reproduction is inherently beneficial in the simulations corresponding to the square points, but not in those corresponding to the triangular points. Note that the benefit assumed in this simulation is insufficient for the twofold cost of male production. The different colors indicate the threshold values of the number of individuals per patch for pesticide application (10: black, 20: red and 40: green). The points with the same color and shape correspond to differences in the dispersal success rate (0.02: left, 0.05: middle and 0.1: right). The arrow indicates the time at which asexual individuals invaded the population.

To explore this possibility, we constructed a simple individual-based simulation model with a finite number of patches (subpopulations) in which the following events occur in order: mating, dispersal, pesticide application and reproduction (see model details in the *Methods* section). Our simulations showed that female-biased sex ratios can evolve and be maintained in populations of sexual individuals, especially after the invasion of asexual individuals (Fig. 3). Although sexual females were given an insufficient advantage (the number of offspring per female was set at 20 for sexual individuals and 15 for asexual individuals) compared with the twofold cost of male production based on the assumption of an equal sex ratio, the asexual individuals still failed to annihilate the sexual individuals because of their female-biased sex ratios (Fig. 3a). It is also difficult for sexual individuals to eradicate asexual individuals because the significant genetic contribution of the males to the next generation of sexual individuals inhibits the evolution of an extremely female-biased sex ratio. As a result, the average sex ratios were stabilized at approximately 0.25 (Fig. 3b, see Supplementary Fig. S2 for the results of the other parameter sets), at which the population growth rate of the sexual individuals was equal to that of the asexual individuals. Our simulations also showed that this mechanism works well only when benefits of sexual reproduction exist (Fig. 4). This result is simply because a no-male condition cannot be achieved by sexual females, as they must produce at least one male for sexual reproduction; thus, the cost of male production never disappears. In addition, although we allowed sexual females to adopt an optimal sex ratio based on LMC theory, in which the proportion of males is (*n* − 1)/2*n* (*n* indicates the number of sexual females in a patch), an LMC strategy did not evolve in our simulations (Supplementary Fig. S3). These results were robust over a range of assessed parameter values (Fig. 4, S2 and S3). Overall, our simulations demonstrated that a female-biased sex ratio can evolve in a sexual strain in response to frequent pesticide applications arising from the invasion of asexual counterpart.

**Figure 5:**
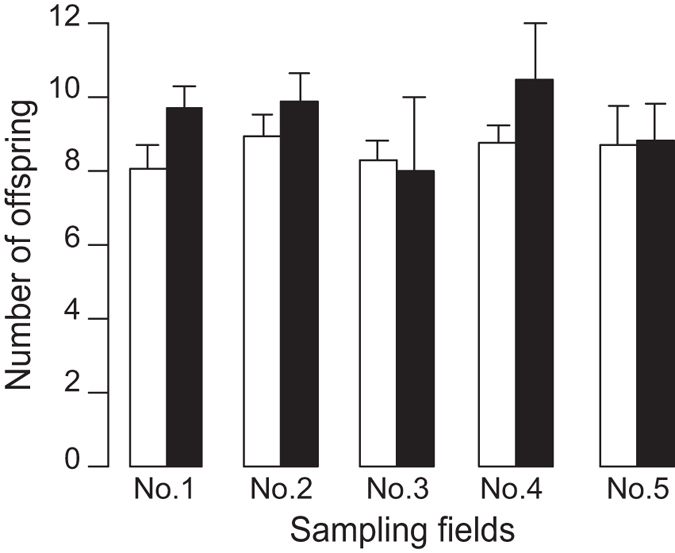
The mean number of offspring produced by sexual and asexual mothers sampled from 5 crop fields. Open and filled bars indicate data corresponding to sexual and asexual mothers, respectively. The bars show the mean values and standard errors.

To determine whether the number of offspring actually differed between the sexual and asexual strains of *T. tabaci*, we measured the reproductive rates of strain sampled from five crop fields (populations) in a laboratory-rearing experiment. The number of offspring did not significantly differ between the strains in the five tested populations (Fig. 5; two-way ANOVA, *n* = 162 mothers produced 1538 offspring; *F*_*1,156*_ = 0.0557, *p* = 0.8138 for strain; *F*_*4,156*_ = 0.5461, *p* = 0.7021 for populations). Thus, the advantages of the sexual strain in their reproductive ability were not detected in this experiment under the constant temperature. In addition, in the sexual strain, we found no male offspring (*n* = 32 mothers produced 224 adult offspring by the end of the experiments), which reflects the female-biased sex ratios observed in the sexual strain.

**Figure 6:**
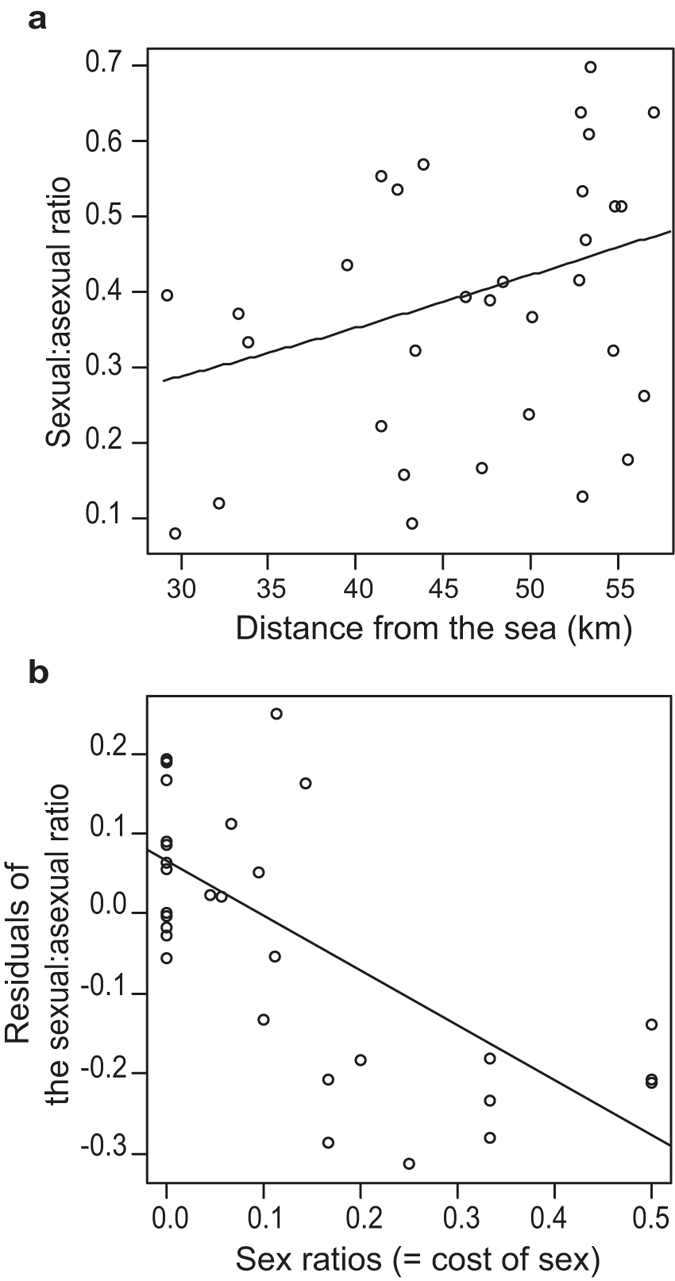
The effect of distance from the sea as a proxy of environmental fluctuation and the cost of male production on the sexual:asexual ratio. (**a**) Sexual strain dominated in inland areas, where the daily temperature shows greater variation than in coastal areas. Each point represents the data corresponding to one population. (**b**) The effect of the sex ratio on the sexual:asexual ratio after removing the effect of distance from the sea. Each the residual value was calculated from the regression line and each point in panel a.

Daily temperature fluctuations, a type of environmental variability, may provide advantages to sexual strain. The distance from the sea (ocean) to each the sampling field was used as an index of the daily temperature fluctuation, as such fluctuations are more variable in inland regions than in coastal regions. This index was positively correlated with the sexual:asexual ratio (Fig. 6a; GLM, *n* = 32; likelihood ratio *x*^*2*^ = 3.9606, *p* = 0.047; Pearson’s product-moment correlation, *r* = 0.356, *p* = 0.045). This analysis may be statistically invalid because of the possibility that geographically close populations may exhibit similar sexual:asexual ratios. Therefore, we analyzed the effect of the geographic distance between populations on differences in the sexual:asexual ratios and confirmed no significant correlation between them (Mantel test using Pearson’s product-moment correlation, *r* = 0.057, *p* = 0.099, based on 10,000 permutations; Supplementary Fig. S4). Thus, a part of advantages of sexual reproduction would be explained by the temperature fluctuation, which is lacked in our experimental condition. Moreover, because the sexual:asexual ratios were at least partially attributed to the environmental variability, we removed the effects of distance to the sea in subsequent analyses. Even after its removal, the sex ratio continued to exert a significant effect on the sexual:asexual ratio (Fig. 6b; linear regression analysis, *n* = 32; *F*_*1,30*_ = 24.57, *p* < 0.001; Pearson’s product-moment correlation, *r* = −0.671, *p* <0.001).

Although there have been a number of studies demonstrating the benefits of sexual reproduction^4^,^5^,^6^,^7^,^8^,^9^, it has not yet been determined whether these benefits are sufficient to compensate for the twofold cost of male production. Few empirical studies have evaluated the cost of male production^11^,^12^,^13^,^14^, but these studies have suggested that there is a cost of producing males. The present study, which attempted to quantify the cost of male production, demonstrated that males are indeed costly, and their cost can be reduced by decreasing the ratio of males to females. Under the conditions where the cost of male production is less than twofold, sexual reproduction can evolve with smaller benefits than previously assumed.

The current system is unique because it includes two types of competition: (*i*) indirect competition between the sexual and asexual strains through pesticide application and (*ii*) competition within the sexual strain in terms of the genetic contribution to next generation of sexual populations. This unique system may cause variations in the sex ratios of sexual strain among populations and therefore enables us to demonstrate a reduction in the cost of male production. Many unresolved questions remain about the current system. For example, is the driving factor underlying the reduced male ratios a result of LMC, pesticide application, or both, or are these reduced ratios caused by other unknown factors? If LMC occurs, the density of thrips will correlate with the sex ratio. Additionally, what is the benefit of sexual reproduction in the current system? By integrating our findings into future studies, the long-standing enigma concerning sexual reproduction can be resolved.

## Materials and Methods

### Empirical experiments

We sampled 1330 adult onion thrips (*T. tabaci*) from 32 onion or leek crop fields in Kuriyama, Naganuma, Nanporo and Kitami city, in Hokkaido prefecture, Japan, from July to September in 2009, 2010 and 2011 (see Supplementary Fig. S5 for a map of the sampling locations). Each crop field was treated as a population. The thrips were dislodged from their host plants onto a white plastic tray by gently shaking the host plant, and then collected using an aspirator. All sampled individuals were sorted into male adult, female adult and larva under a microscope based on the presence or absence of an ovipositor and wings. In the following experiments, we only used adults because the larvae cannot be distinguished their sex. Next, genomic DNA was extracted from the whole body of each the adult using the modified Chelex method^17^. Dried individuals were crushed in 200 μl of a 5% Chelex solution (Bio-Rad, Hercules, CA, USA; 10 mM Tris-HCl, pH 8.0) and 5 μl of proteinase K (TaKaRa, Otsu city, Shiga, Japan; 20 mg/ml), and then incubated at 55 °C for more than 12 hours in 1.5-ml microcentrifuge tubes. Subsequently, the mixtures were boiled at 98 °C for 10 min to inactivate proteinase K. The template DNA were obtained from the aqueous layer next to the Chelex layer.

The reproductive mode of each adult female was determined via polymerase chain reaction with strain-specific primers^16^. The primer set included one primer that encoded a sequence shared by the strain (TCOR, 5′-attgcgtaaattattcctaaaagtcca-3′) and two primers (sexual strain-specific primer TCOS, 5′-aacagcTattctCcttcttttatctC-3′; asexual strain-specific primer TCOC, 5′-gaacagtatatccacctttatcaacG-3′; the capital letters indicate strain-specific nucleotides) that amplified mtDNA fragments of different lengths corresponding to the reproductive modes (261 bp for the asexual strain and 451 bp for the sexual strain). The composition of reaction mixture used for PCR-SSP was as follows: each 10 μl reaction mixture comprised 5 pmol of the consensus primer, 2.5 pmol of each strain-specific primer, 5 μl 2 × MightyAmp Buffer, 0.1 μl MightyAmp DNA Polymerase (TaKaRa; 1.25 U/μl), and 0.5 μl template DNA. PCR-SSP was performed using a 2720 Thermal Cycler (Applied Biosystems, Foster city, CA, USA) with the following temperature cycles: initial denaturation for 2 min at 98 °C, followed by 35 cycles of denaturation and annealing, consisting of 10 sec at 98 °C and 1 min at 60 °C, and a final extension step of 1 min at 68 °C. The fragment lengths of the PCR products were detected via 1% agarose gel electrophoresis using 5 μl of a 100-bp DNA ladder (TaKaRa) with ethidium bromide staining. Then, the sex ratio of each population was calculated by dividing the number of males by the number of sexual individuals, and similarly the sexual:asexual ratio of each population was obtained by dividing the number of sexual individuals by the total number of adults.

After strain identification, up to 20 randomly chosen sexual females were genotyped at nine microsatellite loci to infer phylogenetic relationships among the populations. To amplify these microsatellite loci, a 2720 Thermal Cycler (Applied Biosystems) was used for PCR, which was performed as follows: initial denaturation of 2 min at 96 °C, followed by 35 cycles of denaturation for 5 sec at 98 °C, annealing for 30 sec at temperatures specific to each primer pair, extension for 1 min at 68 °C, and a final extension of 1 min at 68 °C. The primer sequences and annealing temperatures for these microsatellite loci were described previously^23^, in which we confirmed Mendelian inheritance of microsatellite alleles in the sexual strain. Each 10 μl reaction mixture comprised 2 pmol of primers, 5 μl 2 × MightyAmp Buffer, 0.1 μl MightyAmp DNA Polymerase (TaKaRa, Otsu, Japan; 1.25 U/μl), and 1 μl template DNA. A 1-μl aliquot of the PCR product was electrophoresed in tandem with 0.5 μl of Size Standard Kit 400 (Beckman-Coulter, Fullerton, CA, USA) in a CEQ-8000 Genetic Analyzer (Beckman-Coulter). Based on the data obtained regarding allele frequencies, the pairwise genetic distances (F_ST_) between the 32 populations and deviations from Hardy-Weinberg equilibrium for each population were calculated using the program Arlequin, version 3.5.1.2^24^. Then, a neighbor-joining tree was constructed from the pairwise genetic distance matrix using MEGA version 5 software^25^.

Phylogenetically independent contrasts were used to remove the effect of phylogeny from the correlation analysis between the sex ratio and the sexual:asexual ratio. We arranged population pairs without any overlapping branches in the inferred neighbor-joining tree. Thus, 16 population pairs were obtained from the 32 populations (Supplementary Fig. S1). The differences between the sex ratios and the sexual:asexual ratios were calculated for each of the population pairs. This method provides statistically independent values that represent the amount of change on each branch in the phylogeny^22^. Using these differences, we analyzed the evolutionary relationship between the sex ratios and the sexual:asexual ratios.

To explore differences in reproductive rates between the strains, we sampled an additional 50 adult females from each of five crop fields in Nanporo, Hokkaido prefecture, Japan. These females were individually reared in 50-ml centrifuges tubes with 5 ml of molded plaster in a 25 °C chamber under the long-day condition (14 L:10D). A garlic clove with the skin removed was provided for each the female as a food source and oviposition substrate. After 3 days, we removed the adult females from the centrifuge tubes and assessed their reproductive modes using the method described above. The females that died at this point of time were excluded from the following analysis. On the 20th day after the onset of rearing, we sampled and counted all individuals within the centrifuge tubes. At the end of the experiment, we checked the sex of each reproduced adult under a microscope.

All statistics were performed using the computer program R, version 3.1.0. For proportional data (sexual:asexual ratios and sex ratios), we used generalized linear models using the quasi-binomial logit link in the ‘glm’ function and obtained statistical values using the ‘Anova’ function in the ‘car’ package. For the other data (differences in the phylogenetically independent contrast analysis, number of offspring and residuals), we used a general linear model using the ‘lm’ function and the ‘Anova’ function.

### Simulation

To assess the possibility that female-biased sex ratios evolve in sexual populations of *T. tabaci* to compete with their asexual counterparts, we constructed an individual-based model, assuming a haploid population including sexual males and females and asexual females, with discrete generations in 10,000 patches (subpopulations), where the following events occurred within each generation in the following order: mating, dispersal, pesticide application and reproduction.

Mating occurred randomly among sexual individuals within a patch (the males could mate repeatedly, whereas the sexual females mated just once) and was skipped for asexual females, after which all males died, and the females then either remained in their natal patch or dispersed at a dispersal rate of *d*, which was genetically determined by a single locus (0 < *d* < 1). Dispersal was considered successful at a probability of *p* = 0.02, 0.05 or 0.1, and if it failed, the females died. A destination patch was randomly chosen from all of the patches. After dispersal, a pesticide was applied to all individuals in the patches when the number of individuals was greater than a certain threshold value (*t* = 10, 20 or 40). Subsequently, asexual females produced genetically identical daughters except for in cases of mutation, whereas sexually produced offspring inherited one allele from either the mother or father at each locus. For sexual reproduction, the sex ratio (proportion of males among the offspring; *m*) was determined according to two strategies: the first was based on LMC theory^21^ and was represented as (*n*−1)/2*n* (*n* indicates the number of sexual females in a patch), whereas the second was based on the genetic value of locus *g* (0 < *g* < 1). These two strategies of the sexual females were switched by another locus (*L* = 0 or 1; if the value is zero, the female uses her genetic value, whereas if the value is one, she follows the prediction of the LMC theory). We assumed random mutations at a constant probability per generation per locus (i.e., 0.001). If a mutation occurred, the gene was replaced with a random value between 0 and 1 at the locus corresponding to dispersal rate *d* and male ratio *m*, and a value of 0 or 1 was used for the locus corresponding to the switch of sex ratio strategy *L*. After reproduction, all adults died, and the next generation began.

We started the simulations with 10,000 sexual individuals with random genetic values for all loci. After 1,000 generations, 1,000 asexual individuals were introduced into randomly chosen patches. We assessed the effects of three parameters: (*i*) the benefit of sexual reproduction based on the number of offspring per female (20 for sexual individuals and 15 or 20 for asexual individuals); (*ii*) the probability of dispersal success (*p* = 0.02, 0.05 or 0.1); and (*iii*) the threshold of pesticide application (*t* = 10, 20 or 40). For each of the 18 parameter sets, we ran 100 simulations and recorded population dynamics and gene frequencies between reproduction and mating events until either the extinction of sexual or asexual strain occurred, or 10,000 generations had been simulated. We performed all simulations using Visual C++ 2012 and have provided the code for the simulations as a supplementary note online.

## Additional Information

**How to cite this article**: Kobayashi, K. and Hasegawa, E. A female-biased sex ratio reduces the twofold cost of sex. *Sci. Rep.* **6**, 23982; doi: 10.1038/srep23982 (2016).

## Supplementary Material

Supplementary Information
