# Supplementary material for: A female-biased sex ratio reduces the twofold cost of sex
Source: Sci Rep. 2016 Apr 1;6:23982. doi: 10.1038/srep23982 (PMC4817508; doi:10.1038/srep23982)
Supplement: Supplementary Information [file srep23982-s1.pdf]

Supporting Information file

Title: A female-biased sex ratio reduces the twofold cost of sex

Authors: Kazuya Kobayashi[1,*,†], Eisuke Hasegawa[1]

Affiliations:

[1]Laboratory of Animal Ecology, Department of Ecology and Systematics, Graduate School of Agriculture, Hokkaido University, Sapporo 060-8589, Japan

*Correspondence to: kobakaz@kais.kyoto-u.ac.jp

†Laboratory of Insect Ecology, Graduate School of Agriculture, Kyoto University, Oiwake-cho, Kitashirakawa, Sakyo-ku, Kyoto 606-8502, Japan (present address)

Table S1. Summary statistics of population genetics. n_{female} is the number of adult females we genotyped, n_{allele} is the number of observed alleles averaged across the nine loci, H_O is the observed heterozygosity, H_E is the expected heterozygosity under Hardy-Weinberg equilibrium, F_{IS} is the inbreeding coefficients and *p*-value is a result of Fisher's exact test of departure from Hardy-Weinberg equilibrium performed by Arlequin (*26*). H_O, H_E, F_{IS} and *p*-value were estimated for each polymorphic locus and averaged across the loci.

Reproductive mode	Sampling location	n_{female}	n_{allele}	H_O	H_E	F_{IS}	*p*-value
Sexual	Kuriyama 1	15	3.778	0.2593	0.6416	0.5959	0.0362
	Kuriyama 2	2	2.000	0.2778	0.5741	0.5161	0.7036
	Kuriyama 3	20	3.875	0.3313	0.5769	0.4258	0.0906
	Kuriyama 4	3	2.444	0.1482	0.5778	0.7436	0.3476
	Kuriyama 5	2	2.111	0.4000	0.6333	0.3684	0.7333
	Kuriyama 6	8	4.778	0.2778	0.7593	0.6341	0.1377
	Kuriyama 7	20	6.000	0.6000	0.7615	0.2121	0.1557
	Kuriyama 8	20	10.000	0.4056	0.7752	0.4768	0.0298
	Kuriyama 9	2	2.111	0.1875	0.7083	0.7353	0.4163
	Kuriyama 10	8	3.111	0.2361	0.6213	0.6200	0.0888
	Kuriyama 11	3	2.556	0.1482	0.6222	0.7619	0.2599
	Kuriyama 12	12	3.444	0.2315	0.6135	0.6227	0.0407
	Naganuma 1	20	3.889	0.2389	0.5600	0.5734	0.0044
	Nanporo 1	17	4.222	0.2484	0.5391	0.5393	0.0806
	Nanporo 2	10	4.333	0.3222	0.6485	0.5032	0.0628
	Nanporo 3	2	2.000	0.4444	0.5185	0.1429	0.9259
	Nanporo 4	2	1.444	0.3750	0.5417	0.3077	0.8334
	Nanporo 5	20	5.667	0.1167	0.5110	0.7717	0.1489
	Kitami 1	10	5.667	0.4667	0.6866	0.3203	0.3277
	Kitami 2	6	4.000	0.4259	0.6229	0.3162	0.4607
	Kitami 3	19	7.778	0.3860	0.7563	0.4897	0.1394
	Kitami 4	20	5.556	0.2611	0.6187	0.5779	0.0015
	Kitami 5	20	8.333	0.2222	0.7645	0.7093	0.1308
	Kitami 6	20	5.222	0.1889	0.6879	0.7254	0.0055
	Kitami 7	11	5.222	0.1010	0.7653	0.8680	0.0338
	Kitami 8	8	4.889	0.0833	0.7417	0.8876	0.0037
	Kitami 9	20	4.778	0.1222	0.6519	0.8125	0.0042
	Kitami 10	20	9.111	0.2000	0.8274	0.7583	0.0004
	Kitami 11	9	6.667	0.2099	0.8446	0.7515	0.0032
	Kitami 12	12	6.889	0.1944	0.8112	0.7603	0.0034
	Kitami 13	20	8.222	0.1611	0.8100	0.8011	< 0.0001
	Kitami 14	5	3.667	0.1111	0.7136	0.8443	0.0239
Asexual	Kuriyama 8	20	4.556	0.1167	0.5110	0.7717	0.0062
	Nanporo 5	20	6.444	0.1056	0.6812	0.8450	< 0.0001

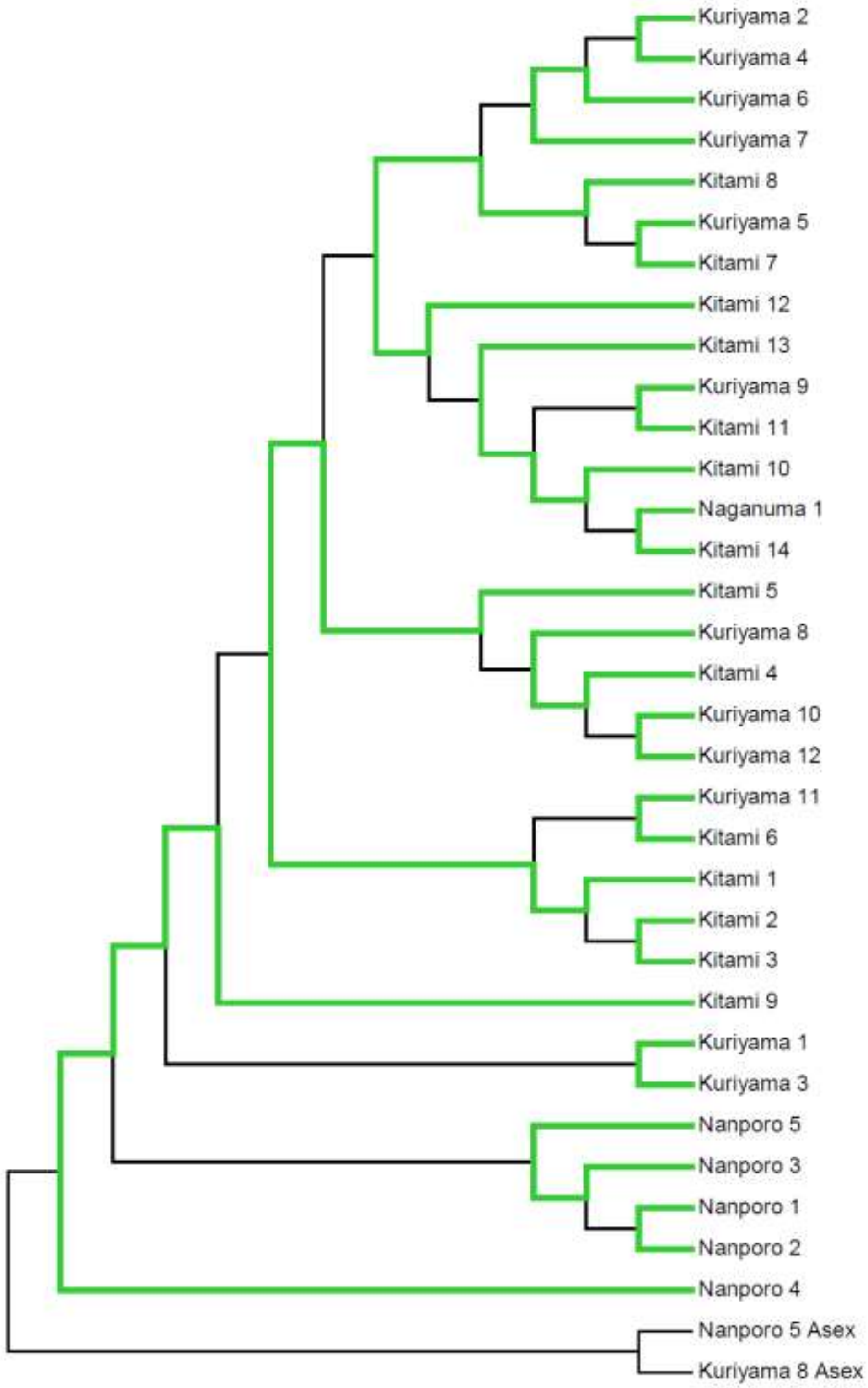

Figure S1. A neighbor-joining tree based on the genetic distances (Fst) among the 32 sexual populations and 2 asexual populations (outgroup). For the phylogenetically independent contrasts analysis, we used 16 population pairs linked by the highlighted branches.

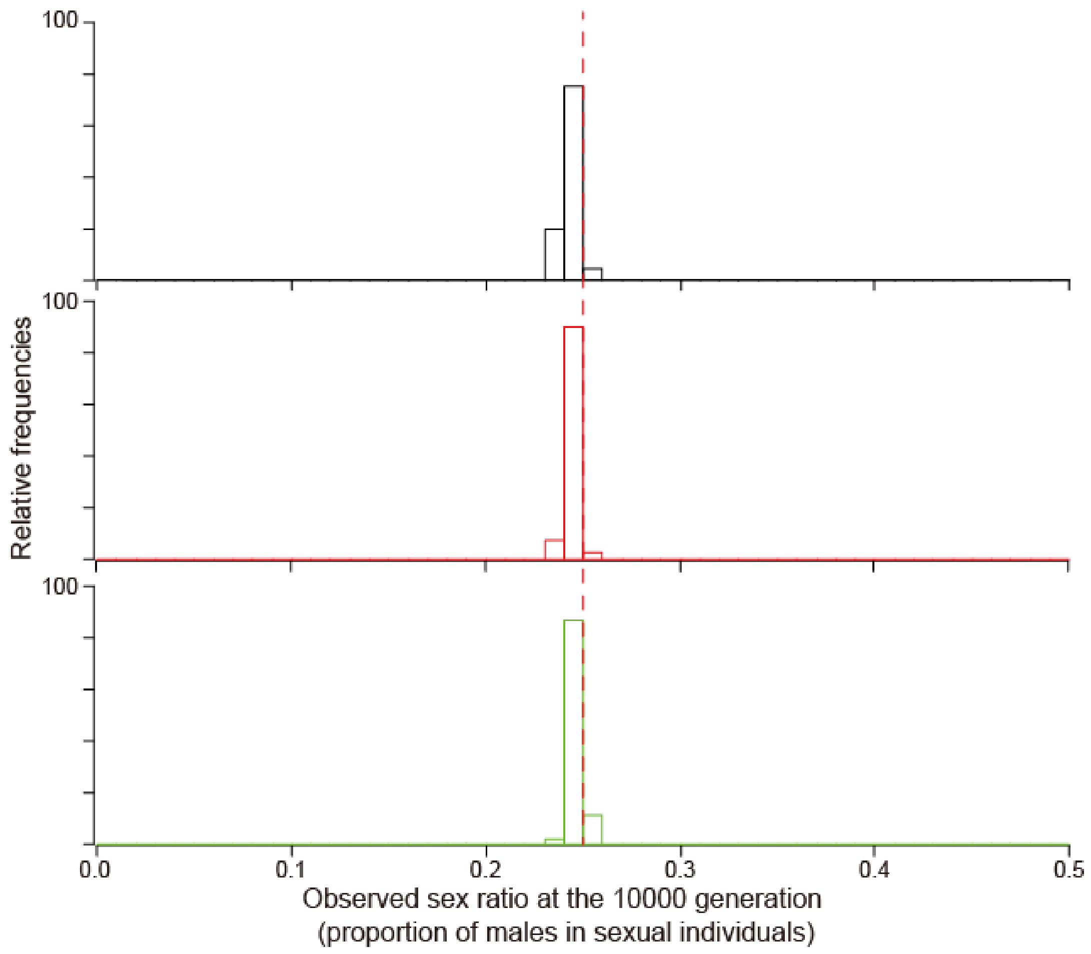

Figure S2. The relative frequency of the observed sex ratio (proportion of males in sexual individuals) at 10,000 generation in sexual individuals coexisting with asexual individuals. Each histogram corresponds to each threshold value of a number of individuals per patch for the pesticide application (10: top black, 20: middle red and 40: bottom green). Red vertical line indicates 0.25 where the population growth rate of sexuals is equal to that of asexuals.

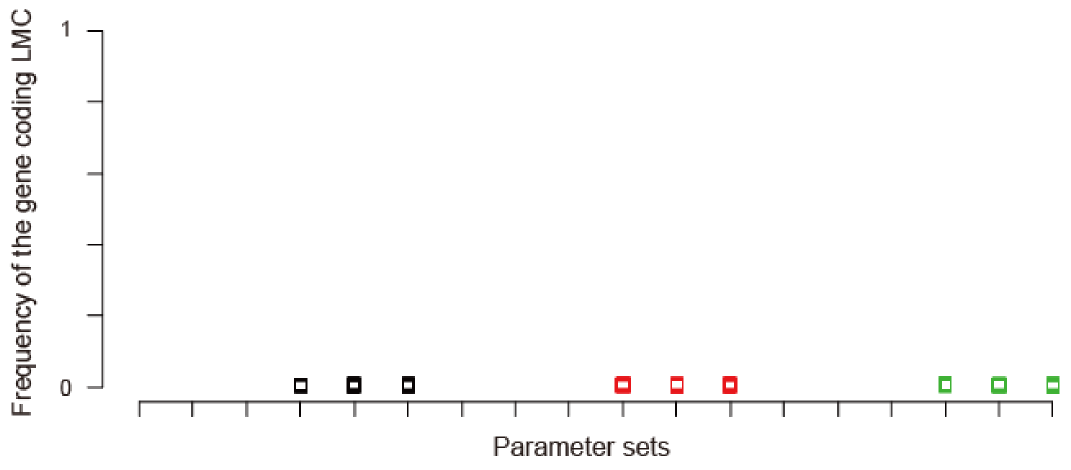

Figure S3. The effect of simulation parameters on the average frequencies of the gene coding the LMC strategy at 10,000 generation in sexual individuals. The order of parameter sets is the same with Fig. 4. Thus, the different colours indicate the threshold values of number of individuals per patch for pesticide application (10: black, 20: red and 40: green). The points with the same colour correspond to differences in dispersal success rate (0.02: left, 0.05: middle and 0.1: right). We cannot represent the data of simulations without the benefit of sex because extinction occurs before the generation reaches 10,000.

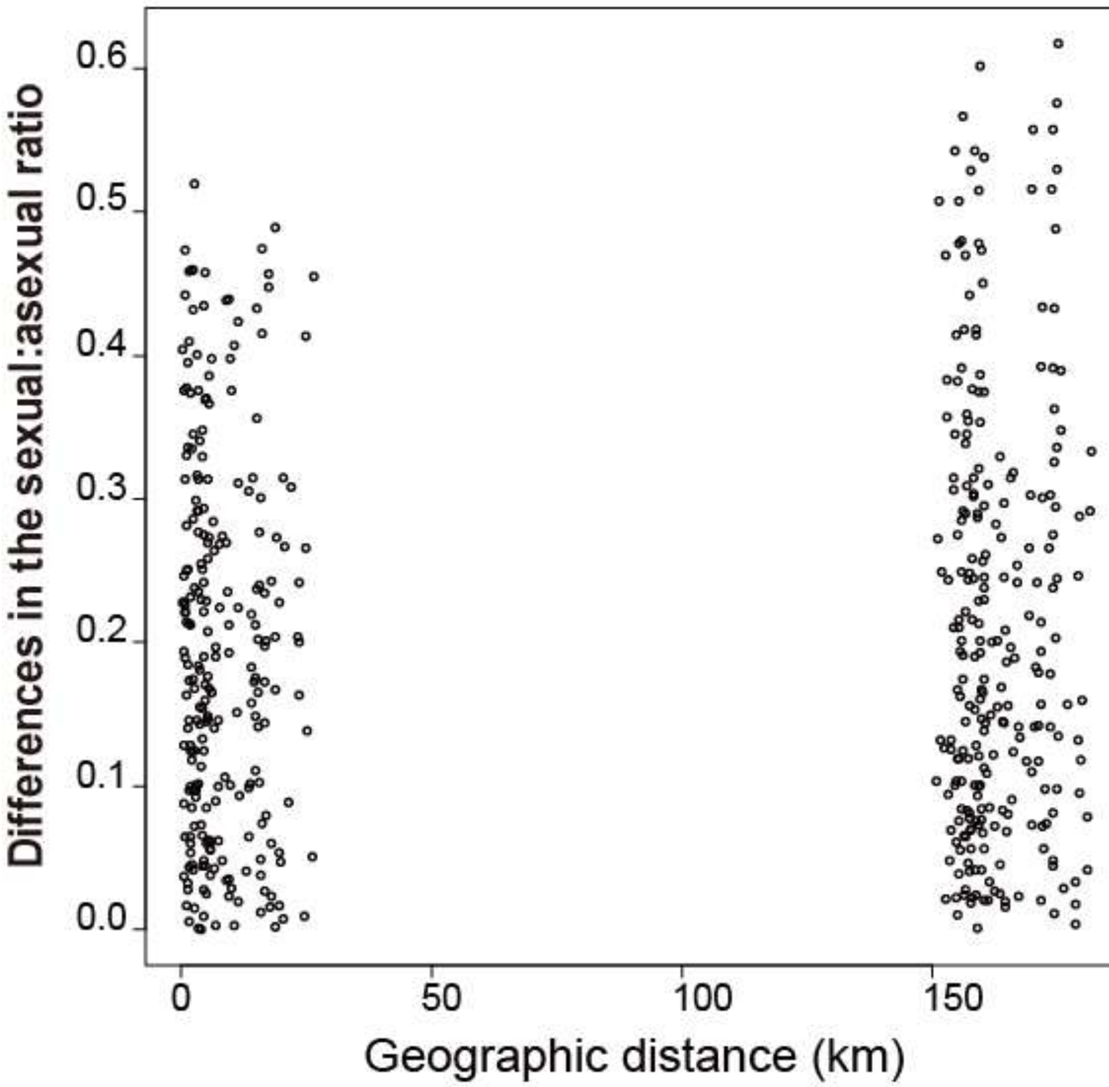

Figure S3. The relationship between geographic distances and differences in the sexual:asexual ratios among the sampling locations, which is used in the Mantel test.

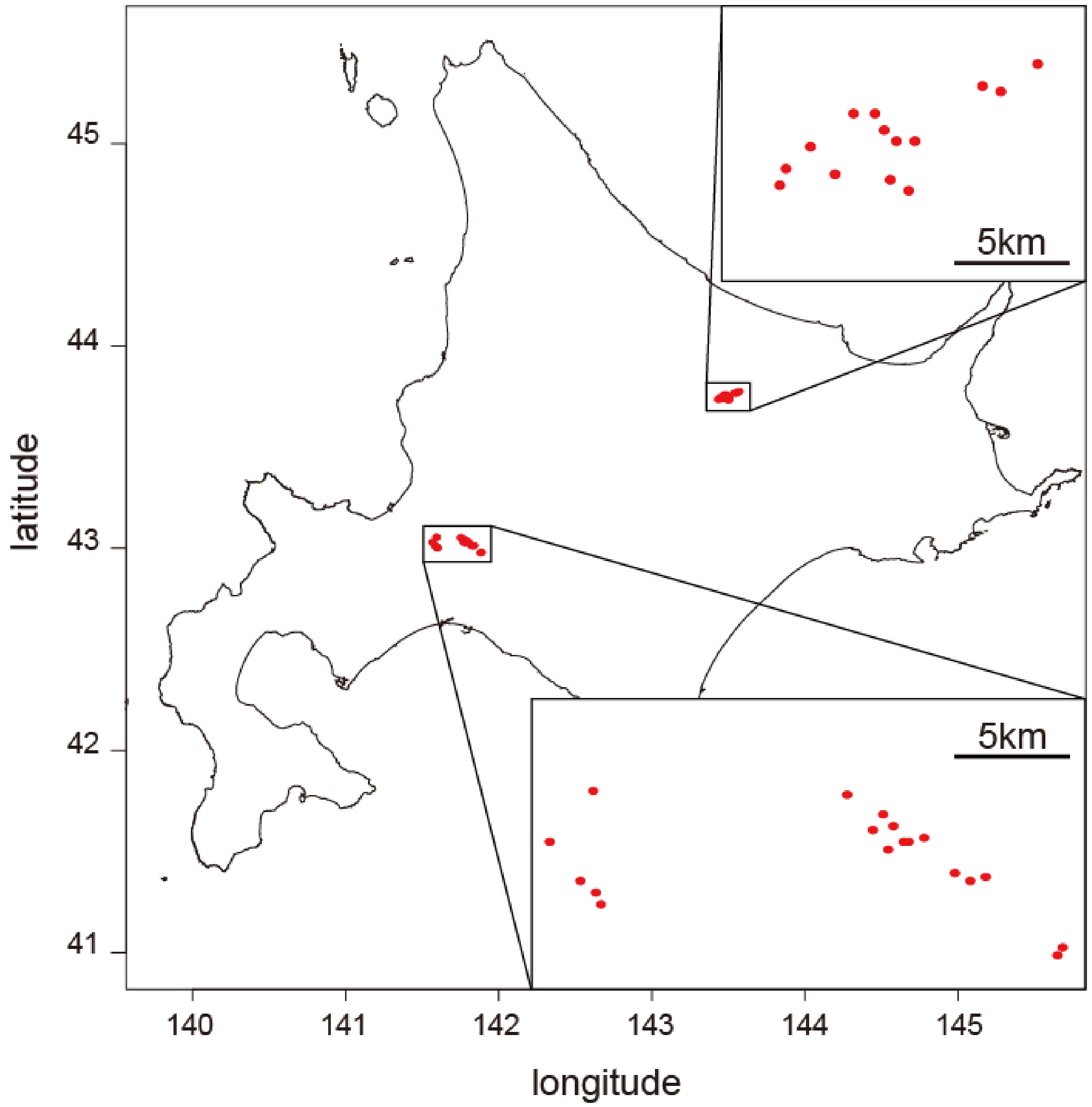

Figure S4. Map of sampling fields. Each red point corresponds to each crop field where we sampled the onion thrips *T. tabaci*. We drew the map using the library maptools (version 0.8-30; http:// cran.r-project.org/package=maptools) in the software R (version 3.1.0; http:// cran.r-project.org/) with the data provided by National Land numerical information (coastal lines data), Ministry of Land, Infrastructure, Transport and Tourism. The data was published in 2006, named as C23-06_01_GML.zip, and downloaded from the web site (http://nlftp.mlit.go.jp/ksj-e/gml/datalist/KsjTmplt-C23.html).

```
// Sex_vs_Asex.cpp
// Microsft Visual Studio Express 2012 for Windows Desktop
// Version 11.0.50727.42

#include "stdafx.h"

#include <random>
#include <iostream>
#include <fstream>
#include <sstream>
#include <string>
#include <time.h>
#include <vector>
#include <numeric>
#include <algorithm>
using namespace std;

//Fixed Parameters
#define mutation 0.001		//mutation rate per loci per generation
#define Timepoint 1000		//invasion of asexual individuals occurs
#define NumIntroduced 1000			//number of introduced asexual individuals


int _tmain()
{
		int extinct;
		bool ex_flag = false;

		//Read parameters from txt file
		int Parameters[8] ={};
		ifstream inputfile("Parameters.txt", ios::in); //read only
		string parameter_name;
		if(inputfile.fail()){
			cout << "Cannot find parameter file(Parameters.txt). press Enter key." << endl;
			getchar();
			return -1;
		}
		int i = 0;
		while(true){//
			if(i % 2 == 0){
				inputfile >> parameter_name;
				if(inputfile.fail()) break;
				cout << parameter_name << " = ";
			} else {
				inputfile >> Parameters[i/2];
				if(inputfile.fail()) break;
				cout << Parameters[i/2] << endl;
			}
			i++;
		}
		inputfile.close();
		cout << "parameter read end" << endl;

		//Setting
		int Num_patch = Parameters[0];							//Number of patch
		int pesticide = Parameters[1];							//Threshold of pesticide application
		int Sreproduction = Parameters[2];						//Number of offspring per sexual female
		int ASreproduction = Parameters[3];						//Number of offspring per asexual female
		long double dispersal_success = long double(Parameters[4]) / 100;		//dispersal success rate
		int initial_population = Parameters[5];						//initial population size
		int Repeat = Parameters[6];							//number of repeat for a parameter set
		int TimeSteps = Parameters[7];							//maximum number of generations
		int Max_pop = Num_patch * pesticide * Sreproduction;

		if(initial_population > Num_patch * pesticide * 0.5){
			cout << "initial_population is too large for the setting. press Enter key." << endl;
			getchar();
			return -1;
		}
		//allocate memory
		vector<int> ReproductiveMode(Max_pop, -1);			//Reproductive Mode(0=asexual; 1=sexual)
		vector<int> sex(Max_pop,-1);					//Sex(0=female; 1=male)
		vector<int> mates(Max_pop,-1);					//id of mates
		vector<int> LMC(Max_pop,-1);					//sex allocation strategy of sexual females(0=genetic; 1=LMC ESS)
		vector<int> location(Max_pop,0);				//location of each individuals
		vector<long double> MaleRatio(Max_pop,-1);			//strategy of sexual females
		vector<long double> DispersalRate(Max_pop,-1);			//dispersal rate of females
		vector<int> patch_population(Num_patch,0);				//# individuals on each patch
		vector<int> patch_males(Num_patch,0);					//# males on each patch
```

```
	vector<int> patch_females(Num_patch,0);						//# females on each patch
	vector<vector<int>> Map_males(Num_patch, vector<int>(pesticide*Sreproduction, -1));	//hold male id on each patch
	vector<int> ReproductiveFemales(Max_pop,-1);
	vector<int> NReproductiveMode(Max_pop, -1);
	vector<int> Nsex(Max_pop,-1);							//
	vector<int> NLMC(Max_pop,-1);							//
	vector<int> Nlocation(Max_pop,0);						//
	vector<long double> NMaleRatio(Max_pop,-1);					//
	vector<long double> NDispersalRate(Max_pop,-1);					//
	vector<int> Npatch_population(Num_patch,0);					//
	vector<int> Npatch_males(Num_patch,0);						//
	vector<int> Npatch_females(Num_patch,0);					//

	vector<long double> ESS(pesticide*2+1,0);
	for(i = 1; i < pesticide*2; ++i){
		ESS[i] = (i-1) / (2*i);
	}

	//allocate memory for output
	int output_data = 7;
	//# asexuals, # sexual females, # sexual males, Dispersal rate of asexuals, Dispersal rate of sexuals, genotype LMC, genotype sexratio
	vector<vector<long double>> output1(TimeSteps, vector<long double>(output_data, 0));	//output of each end of time step
	vector<vector<long double>> output2(Repeat, vector<long double>(output_data+1, 0));	//output of each end of simulation

	//Random function
	random_device rd;						// non-deterministic generator
	mt19937 gen(rd());						// to seed mersenne twister.
	uniform_int_distribution<int> choose_patch(0, (Num_patch-1));	//choose patch
	uniform_int_distribution<int> rbinom(0, 1) ;			//random binomial
	uniform_real_distribution<long double> runif(0, 1) ;		//random uniform



	for(int current_repeat = 0; current_repeat < Repeat; ++current_repeat){
//cout << "---------------------------------Repeat---------------------------------" << endl;
	cout << "repeat #" << current_repeat+1 << " start!" << endl;

	//initialize
	int population = initial_population;
	fill(patch_population.begin(),patch_population.end(),0);	//reset patch population
	fill(patch_males.begin(),patch_males.end(),0);			//reset patch population
	for(i = 0; i < population; ++i){
		int p = choose_patch(gen);
		if(patch_population[p] < pesticide){
			++patch_population[p];
			ReproductiveMode[i] = 1;			//Reproductive Mode(0=asexual; 1=sexual)
			sex[i] = rbinom(gen);				//equal sex ratio for sexuals(0=female; 1=male)
			if(sex[i] == 1){
				Map_males[p][patch_males[p]] = i;
				++patch_males[p];
			}
			LMC[i] = rbinom(gen);						//
			MaleRatio[i] = runif(gen);					//
			DispersalRate[i] = runif(gen);
			location[i] = p;
		}else{
			--i;
		}
	}

	for(int t = 0; t < TimeSteps; ++t){
//cout << "----------------------------generation start----------------------------" << endl;
		//cout << t << " population:" << population << endl;
		if(t == Timepoint){					//invasion of asexual individuals occurs
			for(int n = 0; n < NumIntroduced; ++n){
				ReproductiveMode[population] = 0;
				sex[population] = 0;
				LMC[population] = -1;			//
				MaleRatio[population] = -1;		//
				DispersalRate[population] = runif(gen);
				location[population] = choose_patch(gen);
				++patch_population[location[population]];
				++population;
			}
		}

//cout << "---------------------------------Mating--------------------------------" << endl;
		fill(mates.begin(), mates.end(), -1);				//reset mates
		for(i = 0; i < population; ++i){
```

```
				if(ReproductiveMode[i] == 1 && sex[i] == 0){							//for sexual females
					int p = location[i];
					if(patch_males[p] > 0){								//if males exist in the patch
						mates[i] = Map_males[p][int(patch_males[p] * runif(gen)) ];	//random mating occurs at each patch
					}//
				}
			}//Next patch

//cout << "--------------------------------Dispersal--------------------------------" << endl;
			fill(patch_population.begin(), patch_population.end(), 0);						//reset
			fill(patch_females.begin(), patch_females.end(), 0);							//reset
			fill(ReproductiveFemales.begin(), ReproductiveFemales.end(), -1);					//reset
			int num_ReproductiveFemales = 0;
			for(i = 0; i < population; ++i){
				if(sex[i] == 0){
					if(DispersalRate[i] > runif(gen)){
						if(dispersal_success > runif(gen)){
							location[i] = choose_patch(gen);		//random dispersal(island model)
							ReproductiveFemales[num_ReproductiveFemales] = i;
							++patch_population[ location[i] ];		//counting all females (sexual and asexual)
							++num_ReproductiveFemales;
							patch_females[ location[i] ] += ReproductiveMode[i];	//counting sexual females
						}
					} else {
						ReproductiveFemales[num_ReproductiveFemales] = i;
						++patch_population[ location[i] ];				//counting all females (sexual and asexual)
						++num_ReproductiveFemales;
						patch_females[ location[i] ] += ReproductiveMode[i];	//counting sexual females
					}
				}
			}

//cout << "------------------------------Reproduction-----------------------------" << endl;
			int next_population = 0;
			fill(Npatch_population.begin(), Npatch_population.end(), 0);					//reset
			fill(Npatch_males.begin(), Npatch_males.end(), 0);							//reset
			fill(Npatch_females.begin(), Npatch_females.end(), 0);						//reset
			fill(Nlocation.begin(), Nlocation.end(), -1);								//reset
			fill(NLMC.begin(), NLMC.end(), 0);									//reset
			for(i = 0; i < num_ReproductiveFemales; ++i){
				int id = ReproductiveFemales[i];
				int mid = mates[id];
				int p = location[id];
				if(sex[id]==1){cout << "sex:" << sex[id] << endl;}
				if(patch_population[p] < pesticide){		//reproduction occurs only in the patches where population size is under the threshold
					if(ReproductiveMode[id] == 1 && mid != -1){						//sexual reproduction
						//cout << "sexual: mid is "<< mid << endl;
						for(int n = 0; n < Sreproduction; ++n){
							++Npatch_population[p];
							NReproductiveMode[next_population] = ReproductiveMode[id];
												//ReproductiveMode(0=asexual; 1=sexual)
							if(LMC[id] == 0){
								//LMC[id]=0, sex ratio is genetically determined
								if(MaleRatio[id] > runif(gen)){	//Sex(0=female; 1=male) determined
									//cout <<"male produced"<<endl;
									Nsex[next_population] = 1;			//male produced
									Map_males[p][Npatch_males[p]] = next_population;
									++Npatch_males[p];
								}else{
									//cout <<"female produced"<<endl;
									Nsex[next_population] = 0;			//female produced
									++Npatch_females[p];
								}
							}else{
								//LMC[id]=1, sex ratio is ESS (no variation)
								if(n <= ESS[patch_females[p]] * Sreproduction){
												//the first offspring is always male
									//cout <<"male produced"<<endl;
									Nsex[next_population] = 1;			//male produced
									Map_males[p][Npatch_males[p]] = next_population;
									++Npatch_males[p];
								}else{
									//cout <<"female produced"<<endl;
									Nsex[next_population] = 0;			//female produced
									++Npatch_females[p];
								}
							}
							int r = rbinom(gen);
							NLMC[next_population] = LMC[id] * r + LMC[mid] * (1-r);
```

```
                        // inheritance from mother(r=1) or father(r=0)
                        r = rbinom(gen);
                          NMaleRatio[next_population] = MaleRatio[id] * r + MaleRatio[mid] * (1-r);
                        r = rbinom(gen);
                          NDispersalRate[next_population] = DispersalRate[id] * r + DispersalRate[mid] * (1-r);
                        if(mutation > runif(gen)){ NLMC[next_population] = rbinom(gen);}
                        if(mutation > runif(gen)){ NMaleRatio[next_population] = runif(gen);}
                        if(mutation > runif(gen)){ NDispersalRate[next_population] = runif(gen);}
                        Nlocation[next_population] = p;
                        ++next_population;
                    }
                } else if(ReproductiveMode[id] == 0){                    //asexual reproduction
                    //cout << "asexual:" << id <<", " << next_population << ", " << p << endl;
                    for(int n = 0; n < ASreproduction; ++n){
                        ++Npatch_population[p];
                        NReproductiveMode[next_population] = ReproductiveMode[id];
                          //ReproductiveMode(0=asexual)
                        Nsex[next_population] = ReproductiveMode[id];
                          //asexuals produce only females(=0)
                        NLMC[next_population] = LMC[id];
                        NMaleRatio[next_population] = MaleRatio[id];
                        NDispersalRate[next_population] = DispersalRate[id];
                        if(mutation > runif(gen)){ NDispersalRate[next_population] = runif(gen);}
                        Nlocation[next_population] = p;
                        ++next_population;
                    }
                }
            }
        }
        population = next_population;
        ReproductiveMode = NReproductiveMode;
        sex = Nsex;
        patch_population = Npatch_population;
        patch_males = Npatch_males;
        patch_females = Npatch_females;
        LMC = NLMC;
        MaleRatio = NMaleRatio;
        DispersalRate = NDispersalRate;
        location = Nlocation;

//cout << "--------------------------------Observe----------------------------------" << endl;
    //# asexuals, # sexual females, # sexual males, Dispersal rate of asexuals, Dispersal rate of sexuals, genotype LMC, genotype maleratio
        for(i=0; i < output_data; ++i){
            output1[t][i] = 0;
        }
        for(i = 0; i < population; ++i){
            output1[t][1] += ReproductiveMode[i];                    //counting sexuals including sexual males
            output1[t][2] += sex[i];                                 //counting males
            output1[t][3] += DispersalRate[i] * (1-ReproductiveMode[i]);
            output1[t][4] += DispersalRate[i] * ReproductiveMode[i];
            output1[t][5] += LMC[i] * ReproductiveMode[i];
            output1[t][6] += MaleRatio[i] * ReproductiveMode[i];
        }
        output1[t][0] = population - output1[t][1];
        output1[t][3] /= output1[t][0];
        output1[t][4] /= output1[t][1];
        output1[t][5] /= output1[t][1];
        output1[t][6] /= output1[t][1];
        output1[t][1] -= output1[t][2];                              //calculate sexual females
        //
        if(population < 1){
                ex_flag = true;
                extinct = t;
                break;
        }else if(t > Timepoint){
            if( output1[t][0] < 1 || output1[t][1] < 1 || output1[t][2] < 1){
                ex_flag = true;
                extinct = t;
                break;
            }
        }
    }//end of single generation

//cout << "----------------------------------Output1--------------------------------" << endl;
  char tempDay[32], tempTime[32];
    _strdate_s(tempDay);
    _strtime_s(tempTime);
    cout << "End at " << tempTime << endl;
    //cout << tempDay[6] << tempDay[7] << tempDay[0] << tempDay[1] <<tempDay[3] << tempDay[4] << endl;
```

```
		ostringstream Filepath;
		Filepath << "Rep" << current_repeat << "_20";
		Filepath << tempDay[6] << tempDay[7] << tempDay[0] << tempDay[1] <<tempDay[3] << tempDay[4];
		Filepath << "_" << tempTime[0] << tempTime[1] << tempTime[3] << tempTime[4] << tempTime[6] << tempTime[7] << ".csv";
		ofstream ofs(Filepath.str());

		ofs << "#Asexuals, #SexualFemales, #SexualMales, ADispersalRate, SDispersalRate, LMC, MaleRatio" << endl;
		if(ex_flag){
			cout << "extinction!" << endl;
			for(int t = 0; t < extinct ; ++t){
				ofs << output1[t][0] << ", " << output1[t][1] << ", " << output1[t][2] << ", " << output1[t][3] << ", " << output1[t][4]	<< ", " <<
output1[t][5]	<< ", " << output1[t][6] << endl;
			}
			//copy final condition to output2
			for(i = 0; i < output_data; ++i){
				output2[current_repeat][i] = output1[extinct-1][i];
			}
			output2[current_repeat][output_data] = extinct;
		} else {
			for(int t = 0; t < TimeSteps; ++t){
				ofs << output1[t][0] << ", " << output1[t][1] << ", " << output1[t][2] << ", " << output1[t][3] << ", " << output1[t][4] <<	", " <<
output1[t][5]	<< ", " << output1[t][6] << endl;
			}
			//copy final condition to output2
			for(i = 0; i < output_data; ++i){
				output2[current_repeat][i] = output1[TimeSteps-1][i];
			}
			output2[current_repeat][output_data] = TimeSteps;
		}
		ex_flag = false;
}//end of single repeat

		//cout << "----------------------------------Output2---------------------------------" << endl;
	char tempDay[32], tempTime[32];
		_strdate_s(tempDay);
		_strtime_s(tempTime);
		//cout << tempTime << endl;
		//cout << tempDay[6] << tempDay[7] << tempDay[0] << tempDay[1] <<tempDay[3] << tempDay[4] << endl;
		ostringstream Filepath;
		Filepath << "T" << TimeSteps << "_20";
		Filepath << tempDay[6] << tempDay[7] << tempDay[0] << tempDay[1] <<tempDay[3] << tempDay[4];
		Filepath << "_" << tempTime[0] << tempTime[1] << tempTime[3] << tempTime[4] << tempTime[6] << tempTime[7] << ".csv";
		ofstream ofs(Filepath.str());

		ofs << "#Asexuals, #SexualFemales, #SexualMales, ADispersalRate, SDispersalRate, LMC, MaleRatio, EndTime" << endl;
		for(int t = 0; t < Repeat; ++t){
				ofs << output2[t][0] << ", " << output2[t][1] << ", " << output2[t][2] << ", " << output2[t][3] << ", " << output2[t][4]	<< ", " <<
output2[t][5]	<< ", " << output2[t][6] << ", " << output2[t][7]	<< endl;
		}

		return 0;
}
```
